# Levels of Selected Persistent Organic Pollutants (PCB, PBDE) and Pesticides in Honey Bee Pollen Sampled in Poland

**DOI:** 10.1371/journal.pone.0167487

**Published:** 2016-12-01

**Authors:** Marek Łukasz Roszko, Marta Kamińska, Krystyna Szymczyk, Renata Jędrzejczak

**Affiliations:** Department of Food Analysis, Institute of Agricultural and Food Biotechnology, Rakowiecka, Warsaw, Poland; Institut Sophia Agrobiotech, FRANCE

## Abstract

Chemical plant protection is a commonly discussed factor potentially responsible for decline in pollinators and other beneficial insect populations. Various groups of chemicals including persistent organic pollutants could impact a bee colony’s welfare and are reported to be present in bee tissue and apiary products. The aim of this work was to evaluate the presence of selected persistent organic pollutant and pesticide residues in bee pollen originating from different geographical regions of Poland. Pesticide residues were identified in 60% of tested bee pollen samples. The compounds identified were mainly active ingredients of fungicide preparations. Insecticide active ingredients were up to 30% of the identified residues. The triazole fungicide tebuconazole and the neonicotinoid insecticide thiacloprid were the most frequently found pesticides in pollen. The highest pesticide concentration was determined for prothioconazole (356 μg kg^-1^). Mean concentrations of chlorinated biphenyls–EC6 and EC12 were 194 pg g^-1^ and 74 pg g^-1^, respectively. CB # 28 has the greatest share in the EC6 profile (mean 61 pg g^−1^, 31% contribution). Relatively high contributions were also observed for CBs # 101 (35 pg g^−1^, 18%), # 138 (36 pg g^−1^, 19%) and # 153 (33 pg g^−1^, 17%). CB # 114 and 118 have the highest share in the dioxin-like biphenyls fraction with mean concentrations of 17.6 and 37.6 pg g^−1^ (respectively 23 and 50%). Mean calculated concentrations of 39 polybrominated diphenyl ether congeners (Σ39 BDE) were 20 ± 27.7 pg g^−1^. High variability was observed between maximal and minimal determined concentration values. Individual BDEs were found at different frequencies and varying concentration levels. BDEs # 47, 75 and 99 dominated the profile with average concentrations of 3 pg g^−1^, 3.1 pg g^−1^, and 2.9 pg g^−1^, respectively.

## Introduction

Increasing incidences of the collapse of honeybee colonies and the general decline of other beneficial insects is of great concern. The decrease in the population of pollinators has affected crop production and threatened biodiversity [1–3]. Up to now a number of factors responsible for the decline of pollinators have been identified [4–7]. Several national and regional research programs have been launched to try and understand the key drivers of loss. The losses are multifactorial, with pesticides, habitat loss, pathogens, parasites and environmental factors considered major drivers [8,9]. The relative importance of individual factors has not been fully clarified [10], but there is now evidence that they might act in combination and such interactions may be difficult to predict [5]. The decline of beneficial insects (like bees) is a side effect of the practice of protecting crops against undesirable insects. Bees are unintentionally exposed not only to poisonous insecticides but also to chemicals not exhibiting acute toxicity, such as herbicides or fungicides. Even at low levels, various chemicals may act as stressors on bees and other pollinating insects [11–16]. New classes of systemic insecticides marketed relatively recently are suspected to be responsible for the observed population decline of bee colonies [17–20]. Some special attention has been paid to neonicotinoid insecticides commonly used to protect crops all over the world.

Bees forage in large areas around their hives (range approx. 1.5–10 km and covers 7–100 km^2^) and are, therefore, exposed to any contaminants within this range [21–24]. Even if there is no direct exposure to pesticides (no risk of contact with foliar spray), contaminated pollen or nectar may affect bee health. Bees may also get contaminated from atmospherically derived compounds and particles [25], including industrial chemicals regarded as POPs (persistent organic pollutants). Traces of some legacy organochlorine insecticides [26,27], polychlorinated biphenyls [28], and brominated flame retardants [29] have been found in honey.

According to Cutler et al. (2014) the toxicity of neonicotinoids to bees is unquestionable, but influences of exposure on realistic environmental concentrations are still debated [10]. A number of studies have shown that even low concentrations of various insecticides can impact bee health [30–32]. Pesticides may alter expression of bee larvae genes [33]. Chronic exposure to pesticides may reduce bee resistance to parasitic infestation [34]. Insecticides accumulated in bee tissue may act as stressors for the entire bee colony [35].

At least two other possible routes of exposure to neonicotinoids (NIs) in addition to the contact with spray used for foliar applications have been identified. Often seeds are sold with an application of NIs as a coating, which may be transported throughout the plant. It was proven that this route under realistic field conditions can detrimentally influence entire colonies of pollinators [31,32, 36]. However, some studies performed under field conditions have suggested that bees foraging on plants grown from treated seed do not show negative health effects [37–41]. The other exposure route for NIs and other insecticides is the seed drilling method. Pneumatic drilling of insecticide-treated seeds may produce pesticide-contaminated dust, which could constitute a direct route of bee exposure [13]. In Canada, the 2014 health report on bees noted that a link to NIs and bee death was suspected from the seed drill method of planting [42].

To reduce the risk of exposure of bees and other pollinators to neonicotinoid insecticides, the European Commission restricted application of 3 neonicotinoids (clothianidin, imidacloprid and thiamethoxam) for seed treatment, soil application, and foliar treatment on bee-attractive plants for 2 years [43]. Recently, chemical contaminants in apiculture products were studied by a number of authors [26,28,29]. However, the available data on POPs are still limited. The risk of exposure of bees and other beneficial insects to pesticides still requires more attention to identify key harmful crops and/or agricultural practices. In most cases, such practices include use of pesticides to protect plants, or use by bee keepers to prevent outbreak of diseases in apiaries. Sanchez-Bayo and Goka (2014) using data on toxicity, frequency of exposure and concentrations observed have identified some neonicotinoid insecticides and two organophosphorous insecticides (namely thiamethoxam, imidacloprid, clothianidin, phosmet and chlorpyrifos) as posing the highest threat to bees on a global scale [44]. In addition, honey and bee pollen are popular food products. As such, they should also be evaluated from a food safety point of view.

The aim of this study was to assess levels of various chemical compounds including pesticide residues, polychlorinated biphenyls (PCBs) and polybrominated diphenyl ethers (PBDEs) in bee pollen samples from different regions in Poland

## Materials and Methods

### Chemicals / reagents

Pestiscan/LCMS-grade solvents were exclusively used in this study. Acetonitrile, n-hexane 95%, cyclohexane, chloroform, dichloromethane, methanol, ethyl acetate, toluene, tetrahydrofuran and water were supplied by Sigma-Aldrich (Belefonte, PA, USA). Analytical-grade anhydrous silver nitrate, sodium sulfate, magnesium sulfate, sodium citrate, sodium hydrogen citrate sesquihydrate, sulfuric acid (96%), as well as LCMS-grade acetic acid and ammonium acetate were supplied by Avantor (Gliwice, Poland). Silica gel 60 (0.063–200 mm) and basic alumina were supplied by Merck (Darmstadt, Germany). Procedures for impregnated silica gel preparation were described previously [45]. Envicarb II graphitized carbon, primary secondary amine (PSA) absorbent and bulk C18 were supplied by Sigma-Aldrich (Belefonte, PA, USA). Bio-beads SX-3 were purchased from Bio-Rad (Warsaw, Poland). High purity (>97%) native PCB standards (IUPAC congeners #19, 28, 43, 52, 77, 81, 101, 105, 114, 118, 123, 126, 138, 153, 156, 157, 167, 169, 180, 181, 189, 194) were supplied by Dr Ehrenstorfer (Augsburg, Germany). Native PBDE standards (IUPAC #1, 2, 3, 7, 8, 10, 11, 12, 13, 15, 17, 25, 28, 30, 32, 33, 35, 37, 47, 49, 66, 71, 75, 77, 85, 99, 100, 110, 116, 118, 119, 126, 138, 153, 154, 155, 166, 181, 183, 190) were supplied by AccuStandard (New Haven, CT, USA). ^13^C_12_-labeled PCB standards (IUPAC #77, 81, 105, 114, 118, 123, 126, 156, 157, 167, 169, 189), PBDE standards (IUPAC #28, 47, 99, 100, 154, 183, 209) were supplied by Cambridge Isotope Laboratories (Andover, MA, USA). ^13^C_12_-labeled PCB congeners IUPAC #19 and 181 were used as syringe standards in PCB/PBDE analyses, while triphenyl phosphate (Sigma-Aldrich) was used as an internal standard in pesticide analysis. Pesticide standards were supplied by AccuStandard and Sigma-Aldrich. Water was purified using a HydroLab (Wislina, Poland) water treatment system.

### Test samples

**Fig 1:**
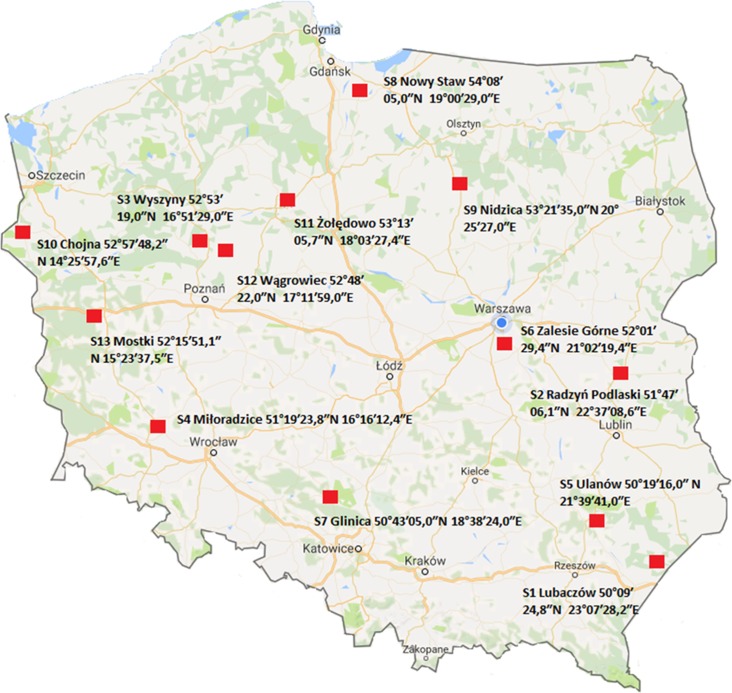
Bee pollen sampling locations.

The concentrations of 161 compounds listed in Table 1 were determined from 53 samples of bee pollen (>50 g each) supplied by beekeepers operating in 13 regions of Poland (shown in Fig 1). The location of each sampling point was recorded. Pollen traps were used to acquire samples. Composite pollen samples from at least three hives from each sampling point have been provided. Samples were acquired between April and May 2015, then refrigerated after collection and later stored at –20°C until the time of analysis. The samples were provided by commercial beekeepers operating on their own land. No additional permissions were required. The study did not involve endangered or protected species.

**Table 1:** Determined compounds, applied quantification technique, and performance of the developed analytical method (spiked samples, n = 15).

N	Compound	Quantified by		LOD	LOQ	R	RSD	N	Compound	Quantified by		LOD	LOQ	R	RSD
		GC/MS	LCMS	[μg kg^-1^]		[%]				GC/MS	LCMS	[μg kg^-1^]		[%]	
1	Aldrin	+		1.7	5.6	76	5	82	Indoxacarb	+	+	0.6	2	85	10
2	Atrazine	+		0.6	2	103	15	83	Ipconazol		+	0.1	0.3	76	5
3	Azoxystrobin	+		0.5	1.8	88	15	84	Iprodion	+		0.9	2.8	64	8
4	Bifenthrin	+		0.2	0.6	93	12	85	Isoproturon		+	1.9	6.2	87	13
5	Bitertanol	+	+	0.3	0.9	86	17	86	Kresoxim methyl	+		0.4	1.4	86	1
6	Boscalid	+		0.1	0.2	76	4	87	Lambda-cyhalothrin	+		0.2	0.6	104	8
7	Bromopropylate	+		0.5	1.8	94	13	88	Lenacil		+	1.3	4.3	90	20
8	Bromucanazol		+	0.2	0.7	81	13	89	Linuron		+	0.5	1.6	90	6
9	Bupirymate	+		0.8	2.6	78	7	90	Malathion	+		1	3.4	84	15
10	Captan	+		2.8	9.2	78	18	91	Mecarbam	+		1.4	4.8	116	5
11	Carbendazim		+	0.6	2.1	88	16	92	Mepanipyrim		+	0.6	2	113	10
12	Carboxin	+		0.7	2.4	83	11	93	Metalaxyl	+		0.9	3.1	75	4
13	Chloridazone	+	+	1	3.2	92	18	94	Metamitron		+	1.5	5.1	79	7
14	Chlorothalonil	+		0.4	1.5	30	22	95	Metconazol		+	0.1	0.3	74	6
15	Chlorphenvinfos	+		0.9	2.9	77	7	96	Methamidophos	+	+	2.7	9.1	89	2
16	Chlorpiryphos	+		1	3.2	93	9	97	Methidathion	+		4.7	16	79	6
17	Chlorpiryphos-methyl	+		0.4	1.4	75	7	98	Methiocarb	+	+	4.2	14	86	14
18	Chlortraniliprole		+	0.3	0.9	86	9	99	Methoxyfenozide		+	0.5	1.7	84	12
19	Cis-chlordan	+		0.9	3.1	102	11	100	Metribuzin	+		0.8	2.8	69	7
20	Clethodim		+	0.8	2.7	89	19	101	Metsulfuron methyl		+	0.6	1.9	84	7
21	Clothianidin		+	0.6	2	86	14	102	Myclobutanil	+		0.8	2.8	86	4
22	Cycloxidime	+		1.6	5.4	79	13	103	Naprapamide		+	0.2	0.6	80	10
23	Cyfluthrin	+		1.3	4.5	103	9	104	Nicosulfuron		+	1.8	5.9	84	7
24	Cymoxanil		+	1.2	3.9	107	10	105	Nitrophen	+		1.5	4.9	73	11
25	Cypermethrin	+		0.8	2.5	113	11	106	Nuarimol		+	0.3	0.9	86	7
26	Cyproconazol		+	0.2	0.7	81	14	107	Oxadixil	+		1.5	4.9	77	14
27	Cyprodinil	+		0.2	0.6	76	8	108	Paclobutrazol		+	0.1	0.4	86	14
28	DDD	+		0.7	2.4	73	10	109	Parathinon-methyl	+		1.3	4.4	77	8
29	DDE	+		0.5	1.7	79	9	110	Parathion	+		2.2	7.3	73	4
30	DDT-op	+		0.5	1.8	75	11	111	Penconazol		+	0.1	0.4	84	6
31	DDVP	+		0.4	1.3	66	20	112	Pencycuron	+	+	3.6	12	93	3
32	Deltamethrin	+		0.5	1.8	105	12	113	Pendimethalin	+		0.9	3.1	75	8
33	Desmedipham		+	0.1	0.5	79	14	114	Permethrin	+		0.2	0.7	95	9
34	Diazinon	+		0.9	2.8	75	7	115	Phenmedipham		+	0.1	0.5	87	16
35	Dichlofluanide	+		0.5	1.8	71	12	116	Phosalone	+		0.9	3	74	4
36	Diclobutrazol		+	0.1	0.3	79	5	117	Phosmet	+		0.4	1.2	75	2
37	Dieldrin	+		3.2	11	73	9	118	Phosphamidone	+		0.8	2.5	79	9
38	Difenoconazol		+	0.1	0.4	76	7	119	Picoxystrobine	+		0.6	2	89	11
39	Difenyloamine	+		0.2	0.8	76	5	120	Pirimethanil	+		0.2	0.6	70	7
40	Dimethoate	+	+	0.3	1.2	97	18	121	Pirimicarb	+		0.3	0.9	47	20
41	Diniconazol		+	0.1	0.3	81	17	122	Pirimiphos-methyl	+		0.7	2.4	85	13
42	DMDT-op	+		0.5	1.7	83	9	123	Procymidone	+		0.5	1.7	85	15
43	DMDT-pp	+		0.7	2.2	75	12	124	Promethrin		+	0.4	1.2	83	9
44	Endosulfan alfa	+		4.5	15	109	10	125	Propachlor	+		0.4	1.4	74	4
45	Endosulfan beta	+		3.7	12	79	11	126	Propaquizafop		+	0.4	1.4	78	3
46	Endosulfan sulfate	+		2.4	8.1	84	10	127	Propargit	+		0.5	1.8	85	6
47	Endrin	+		3.2	11	74	9	128	Propiconazole	+	+	0.9	2.9	86	18
48	Epoxyconazol		+	0.1	0.4	90	1	129	Propoxur	+		0.2	0.5	81	6
49	Esfenvalerate	+		0.3	0.9	82	8	130	Propyzamide	+		0.4	1.3	78	13
50	Etaconazol		+	0.1	0.5	81	13	131	Prothioconazole		+	2.5	8.4	82	4
51	Ethion	+		0.7	2.4	91	17	132	Pyraclostrobin	+	+	1.3	4.2	107	10
52	Ethirimol		+	0.2	0.6	89	12	133	Pyriproxifen	+		0.3	0.9	76	6
53	Etophumesate	+	+	0.5	1.5	100	20	134	Quinmerac		+	0.6	2.1	102	11
54	Etoxazol		+	0	0.2	79	14	135	Quinoxyfen	+	+	0.5	1.5	74	15
55	Etrimfos	+		1.4	4.6	79	12	136	Quintezone	+		1.8	5.9	76	3
56	Fenamidon	+	+	0.5	1.8	61	23	137	Quizalofop-p-ethyl	+		0.9	3	82	2
57	Fenarimol	+	+	0.9	2.9	89	16	138	Quizalofop-P-tefuryl		+	1.4	4.6	98	17
58	Fenazaquin		+	0.9	3	77	7	139	Simzine	+		1	3.4	85	9
59	Fenbuconazol		+	0.2	0.6	92	10	140	Spinosad A		+	0.3	0.9	68	15
60	Fenhexamid	+	+	0.2	0.7	78	13	141	Spinosad D		+	1	3.3	66	18
61	Fenitrothion	+		1.7	5.6	73	6	142	Tau-fluvalinate	+		4.5	15	83	12
62	Fenpropimorph	+		0.4	1.3	87	14	143	Tebuconazole	+	+	1	3.4	81	9
63	Fenvalerate	+		0.5	1.6	79	12	144	Teflutrin	+		1.7	5.7	88	9
64	Fipronil		+	0.3	0.8	81	16	145	Tepraloxidime	+	+	0.4	1.5	83	10
65	Fluazifop-p-butyl	+		0.5	1.6	85	11	146	Tetraconazol		+	0.1	0.4	93	2
66	Fludioxonil	+		0.5	1.8	79	2	147	Tetradifon	+		0.8	2.7	79	5
67	Fluquinconazol		+	0.5	1.6	86	15	148	Thiabendazole		+	2.4	8.1	79	18
68	Flusilazol	+	+	0.3	0.9	85	9	149	Thiacloprid		+	0.3	0.8	112	6
69	Flutriafol		+	0.2	0.6	81	9	150	Thiametoxam	+	+	2.7	8.9	87	13
70	Folpet	+		2.5	8.4	75	2	151	Tiophanat methyl		+	1.4	4.7	77	9
71	Fuberidazole		+	1.8	6.1	82	14	152	Tolyfluanid	+		0.4	1.4	78	8
72	HCB	+		0.4	1.5	73	9	153	Trans-chlordan	+		0.8	2.7	79	11
73	HCH-alfa	+		2.2	7.3	79	8	154	Triadimenol	+	+	0.7	2.5	83	13
74	HCH-beta	+		2.1	6.9	82	11	155	Triazophos	+		0.9	3	105	7
75	HCH-delta	+		1.1	3.8	79	11	156	Trifloxystrobin	+		0.7	2.5	94	10
76	HCH-gamma	+		1.3	4.4	73	8	157	Triflumizole		+	0.1	0.2	77	3
77	Heptachlor	+		1.5	4.9	73	11	158	Trifluralin	+		0.7	2.2	78	9
78	Heptachlor-epoxide	+		1.3	4.2	81	13	159	Triflusulforon methyl		+	1.3	4.3	87	14
79	Hexaconazol		+	0.1	0.3	76	7	160	Triticonazol		+	0.1	0.4	91	17
80	Imazalil		+	0.2	0.6	76	7	161	Vinclozolin	+		1.3	4.4	80	9
81	Imidacloprid		+	1.1	3.7	86	13								

### Sample preparation

#### Pesticides

Pesticide determinations were performed under ISO-17025:2005 accredited procedures based on the EN-15662:2008 method. Briefly, 5 g pollen samples were placed in 50 ml polypropylene centrifuge tubes and mixed with 10 ml of ACN:water (1:1) mixture, spiked with internal standard (250 ng) and cooled in a refrigerator for 20 minutes. Samples were subsequently homogenized, mixed with 4 g of anhydrous magnesium sulfate, 1 g of sodium chloride, 1 g of sodium citrate and 0.5 g of sodium hydrogen citrate sesquihydrate, immediately shaken to avoid magnesium citrate caking and then centrifuged (15000 x G). The organic layer was transferred into a 15 ml polypropylene centrifuge tube and left in a refrigerator overnight to freeze out excessive lipids and residual water. The solution was filtered through glass wool. Two milliliters of organic solution were transferred into a 2 ml polypropylene Eppendorf tube and shaken with 300 mg of magnesium sulfate, 60 mg of PSA and 50 mg of C18, filtered through a 0.45 μm nylon syringe filter and acidified with 30 μl of a formic acid: ACN solution (5:95). The extract was then split into two equal portions blown down under a gentle stream of nitrogen and reconstituted using 250 μl of ethyl acetate (GC) or a water: methanol solution (20:80) (LC) and analyzed using gas and liquid chromatography coupled to mass spectrometry. All determinations were performed in duplicate. Every batch of samples included spiked samples, and matrix matched calibration standards. For determination of some pesticides with LCMS, additional cleanup was required using the same final cleanup as done previously without PSA. The latter were analyzed simultaneously within the sample batch.

#### PCBs / PBDEs

The analytical procedure was based on a slightly modified procedure reported previously [46]. Briefly, 20 g of sample were spiked with ^13^C_12_-labeled internal standards of PCB and PBDEs (50 pg each). Samples were homogenized with 50 ml of chloroform: methanol mixture (2: 1) and sonicated. The solution was then filtered and poured into a separator funnel and mixed with 100 ml of deionized water. The organic phase was transferred into around bottom flask evaporated using a rotary evaporator operated at 40°C. Lipid content was determined gravimetrically. The organic extract was re-dissolved in 75 ml of n-hexane and mixed with 25 ml of sulfuric acid. Samples were left to hydrolyze for at least 8 h. The organic extract was subsequently passed through a chromatographic column (20 mm I.D.) containing 5 g of anhydrous sodium sulphate and eluted with an additional 20 ml of n-hexane. The solution was subsequently evaporated to dryness and re-dissolved in 2 ml of the mobile phase which was composed of a dichloromethane: cyclohexane mixture (1:1) and injected into a GPC system to perform separation on the 500x10 (Omnifit, Cambridge,UK) glass column filled with BioBeads SX-3 styrene-di-vinylo-benzene-based resin.

A 2 ml sample loop and an LDC Analytical Constametric III HPLC pump (Riviera Beach,FL, USA) were used. Elution was performed at the 1 ml min^-1^ flow rate, fraction between 25 and 50 ml was collected. Final clean-up and fractionation were performed using four open tubular liquid chromatography columns (20 mm I.D.).

The first column was filled (bottom to top) with anhydrous sodium sulphate (500 mg), silica gel (250 mg), KOH-impregnated silica gel (10% w/w, 1.5 g), silica gel (250 mg), H_2_SO_4_-impregnated silica gel (2 g, 44% w/w), silica gel (100 mg) and anhydrous sodium sulphate (500 mg). The column was connected in series with a second column filled (bottom to top) with anhydrous sodium sulphate (500 mg) and basic alumina (2 g). The alumina was activated at 600°C overnight before use. The GPC-cleaned-up sample was evaporated to dryness and transferred with 2x2 ml portions of n-hexane to the top of the first column. Columns were previously pre-washed with n-hexane. The columns were washed with 50 ml of n-hexane; then, the second column was washed with 50 ml of a n-hexane: dichloromethane mixture (1:1). The elutant was evaporated to dryness and transferred to the top of the third column filled (bottom to top) with anhydrous sodium sulphate (500 mg) and AgNO_3_-impregnated silica gel (2.0 g). The column was connected to the next column containing 2 g of of envicarb graphitized carbon black dispersed over 8 g of silica gel and covered with anhydrous sodium sulphate. The columns were first eluted with 30 ml of a dichloromethane: n-hexane mixture (2: 98) and then disconnected. The first column was eluted with 30 ml of a dichloromethane: n-hexane mixture (1: 1) (fraction containing PBDEs), while the carbon column was eluted with additional 65 ml of a toluene: n- hexane mixture (5:95) (the fraction containing mono-, di-, tri-, tetra-ortho-PCBs) and then with 65 ml of toluene (fraction containing non-ortho substituted PCBs). The system composed of carbon columns was connected to a pressurized nitrogen gas cylinder (N5.0) to apply a positive pressure on the column head and to accelerate the elution process. The obtained fractions were evaporated to dryness, transferred with n-hexane into 4 ml reaction vials, spiked with syringe standards (^13^C_12_-labelled PCB19 and 181) and blown down under a gentle stream of nitrogen to a final volume between 50–300 μl. The samples were transferred into auto-sampler vials and submitted for GC/MS/MS analysis. Three separate chromatographic runs corresponded to the isolated fractions.

### GC/MS analyses

#### Pesticides

An Agilent (Santa Clara, Ca, USA) 7890A gas chromatograph coupled to a quadruple mass spectrometer 5975C was used for pesticides determination. Two microliters of a clean extract was introduced through a purged splitless (30 PSI) injection at 280°C. Chromatographic separations were performed on a 30 m x 0.25 mm x 0.25 mm Rtx-5 MS 5%-phenyl- fused-silica capillary column (Restek, Bellefonte, PA, USA) connected to a 5 m x 0.25 mm guard column / retention gap (Restek). Helium was used as the carrier gas at constant flow of 1.2 ml min^-1^. The following temperature program was used: 40°C for 1 min, 25°C min^-1^ to 150°C, 1°C min^-1^ to 190°C, 10°C min^-1^ to 300°C for 6 min. The total run time was 62.4 min. The mass spectrometer transfer line, ion source, and quadruple were kept at 300°C, 250°C, and 150°C, respectively. Masses were calibrated against perfluorotributylamine (FC-43) in electron-impact positive ionization mode in line with manufacturer's recommendations. The electron multiplier bias was 1882 V. Data were acquired simultaneously in the ion monitoring/scan modes.

#### PCBs / PBDEs

The instrument setup was identical as reported previously [46]. Briefly, a Thermo-Finningan Trace GC Ultra gas chromatograph (Austin, TX, USA) connected via a heated transfer line with a Polaris Q low-resolution ion-trap mass spectrometer (Austin, TX, USA) was equipped with a Programmable Temperature Vaporizer (PTV) based injector, TriPlus Autosampler (Austin, TX, USA). Chromatographic separations of PCB and PBDEs were performed on a 60 m x 0.25 mm x 0.25 mm Rtx-5 MS 5%-phenyl- fused-silica capillary column (Restek) connected via a Vu2 Union connector (Restek) to a 5 m x 0.25 mm guard column / retention gap (Restek). In all cases helium was used as a carrier gas at a constant flow of 1.5 ml min^-1^. Instrument operational parameters used in the analyses of PCB, PBDE were published previously [46]. Samples were introduced via PTV injector operated in solvent split mode as reported previously. Forty microliters of concentrated extract was introduced (300°C transfer phase). The mass spectrometer transfer line and ion source were kept at 320°C and 300°C, respectively. Masses were calibrated against perfluorotributylamine (FC-43) in electron-impact positive ionization mode in line with manufacturer's recommendations. Multiplier bias was 1475 V, and the automatic gain control was set to 15. The mass spectrometer was operated in the MS/MS mode. Helium was used as the ion-trap dumping gas (flow 1.7 ml min^-1^) to increase ion cooling efficiency. Injection wave form scaling was set to 0.75. Emission current was 350 mA. Excitation voltages necessary to optimize ion yield, isolation/excitation time were determined experimentally by repeated analyses of standard solutions.

### LC/MS analyses

An Acquity H-Class ultra-high performance liquid chromatograph coupled to a LCQ Premiere XE time-of-flight high resolution mass spectrometer (Waters, Milford, MA, USA) was used. Chromatographic separations were performed on a non-porous (Cortecs) 100 mm x 2.1 mm x 1.6 μm C18 column (Waters). An inline filter was placed in front of the chromatographic column instead of a pre-column. Mobile phase composed of water-methanol 90:10(A) and methanol-water 90:10(B), each with 4 mM of ammonium acetate and 0.2% acetic acid, was applied at a flow rate of 300 μl min^-1^ and at the following A:B(%) gradient: 0–2 min, 100:0; 2–3 min, 50:50; 3–6 min, 50:50; 6–22 min, 0:100; 22–23 min 0:100; 23–25 min, 100:0; 25–28, 100:0. Five microliters of the extract was introduced into the system. Experiments were performed using electro spray positive ionization. Ion source and de-solvation temperatures were 150°C and 350°C, respectively. The nebulizing gas (nitrogen) flow rate was 750 L h^-1^ while the cone gas flow rate was 40 l min^-1^. The capillary voltage was 3,200 V and the ion optics was operated in V mode. The mass spectrometer was calibrated using a leucine encephalin standard solution. The nominal mass resolution during experiments was not lower than 6,000 m/z values, corresponding to the (M+H)^+^ pseudo-molecular ions and (M+NH_4_)^+^ and (M+CH_3_OH+H)^+^ adducts were used for calculations. Linearity (as evaluated with standard solutions) was in the 10^3^ range, typical for instruments of that type.

### Data analysis

Enhanced Chemstation E version 02.01.1177 and MassLynx 4.1 software was used to acquire and analyze data. Individual results are given as a mean value of two parallel determinations (±1 SD, if given). The results were statistically assessed with a Statistica 9.0 software suite. Kruskal-Wallis non-parametric tests were used to assess statistical significance of differences between the determined values. The obtained data were also statistically assessed using the Principal Component Analysis method

Statistical parameters of both methods used to determine PCBs and PBDEs have been discussed in detail in our previous papers [45–47]. Basic parameters (recovery rate, repeatability, etc.) used for pesticide analysis were evaluated using spiked samples made in-house. The evaluated recovery values, recovery relative standard deviation (RSD), limits of detection (LOD) and limits of quantification (LOQ) are shown in Table 1. For the majority of compounds determined in bee pollen samples, recovery rates ranged from 70–120%; the relative standard deviation was below 20%. Lower recovery rates were observed only in 7 of 161 cases.

## Results and Discussion

### Pesticides

Twenty-nine pesticide compounds were found in 60% of 53 tested bee pollen samples. In most cases (68% of all positive samples), more than one active compound was identified. A summary of pesticides determined in the samples is given in Table 2.

**Table 2:** Breakdown of pesticides found in the analyzed bee pollen samples (sorted in descending order by the number of positive samples).

Item	Compound	Positive samples		Average	Min	Max
		[n]	[%]	[μg kg^-1^]		
1	Tebuconazole	11	20.8%	29.8	4.0	64.6
2	Thiacloprid	10	18.9%	61.3	3.3	136.0
3	Chlorpyrifos	7	13.2%	15.7	4.4	40.1
4	Propiconazole	6	11.3%	23.4	4.2	48.3
5	DDE	5	9.4%	7.4	5.5	9.9
6	Cyprodinil	5	9.4%	16.3	4.8	23.7
7	Pirimethanil	3	5.7%	17.1	12.6	23.7
8	Boscalid	3	5.7%	19.2	7.6	26.6
9	Fenhexamid	3	5.7%	18.2	13.8	23.8
10	Tetraconazole	3	5.7%	17.7	14.0	21.8
11	Prothioconazole	3	5.7%	181.9	56.6	356.7
12	Tau-fluvalinate	2	3.8%	20.6	13.5	27.7
13	Deltamethrin	2	3.8%	46.7	23.7	69.8
14	Propyzamide	2	3.8%	17.7	12.0	23.3
15	Triadimenol	2	3.8%	12.6	12.6	12.7
16	Flusilazol	2	3.8%	10.4	8.2	12.7
17	Kresoxim methyl	2	3.8%	8.3	7.2	9.3
18	Propargit	2	3.8%	16.0	9.4	22.7
19	Azoxystrobin	2	3.8%	9.4	6.1	12.7
20	Cymoxanil	2	3.8%	13.3	4.4	22.2
21	Bifenthrin	1	1.9%	10.7		
22	Chlorothalonil	1	1.9%	7.3		
23	Metalaxyl	1	1.9%	12.9		
24	Fludioxonil	1	1.9%	11.5		
25	Bitertanol	1	1.9%	36.8		
26	Imidacloprid	1	1.9%	3.1		
27	Metamitron	1	1.9%	62.2		
28	Clethodim	1	1.9%	21.4		
29	Difenoconazol	1	1.9%	5.9		

Literature data varies depending on the number of the studied pesticides, sample matrix/origin or different usage of individual products in different countries, etc. Balayiannis and Balayiannis (2008) reported that residues of organophosphate insecticides were found in 56% of their samples of honey produced by bees foraging mostly in Greek citrus groves [48]. Mullin et al. (2010) reported that more than 92% of 749 analyzed apiculture samples (wax, pollen, bees) from US contained detectable residues of two or more pesticides, mostly compounds used to control pests in beehives (veterinary products) and various classes of agricultural products [49]. The reported concentrations ranged up to tens of mg kg^-1^ of the sample. Pohorecka et al. (2012) studied pollen/nectar from Poland and found that residues of two or more different neonicotinoid insecticides were detected in more than 50% of all analyzed samples [38]. These data are generally in line with the results of this study.

**Fig 2:**
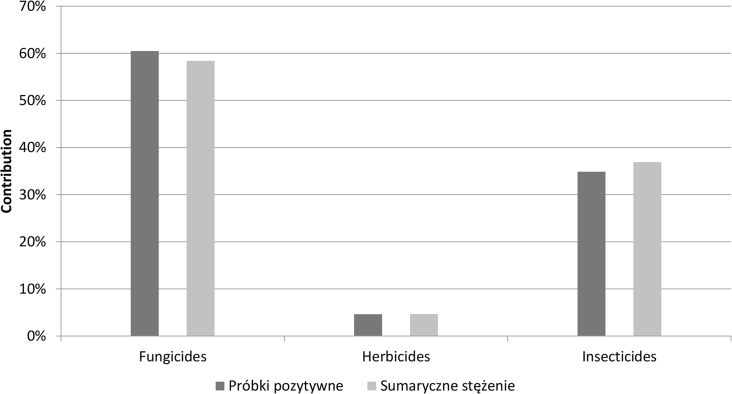
Groups of pesticides identified in the analyzed samples.

The contribution of various groups of pesticides (fungicides, herbicides, insecticides) found in positive samples identified in this study is shown in Fig 2. The proportion was 60:5:35, respectively.

Both the literature and this study indicate that bee pollen can be contaminated with various classes of pesticides. Compounds most frequently found in our samples included tebuconazole (a triazole-class fungicide), thiacloprid (a neonicotinoid insecticide), and chlorpyrifos (20.8%, 18.9% and 13% samples, respectively). Prothioconazol (another triazole-class fungicide) was found at the highest absolute concentration of 356.7 μg g^-1^. Mullin et al. (2010) also found tebuconazole and thiacloprid at similar concentration levels (1.7–115 μg kg^-1^, 0.9–34 μg kg^-1^, respectively), but in a relatively limited number of their bee pollen samples (from US) [49]. Chauzat et al. (2006) found residues of imidacloprid in more than 69% of their bee pollen samples (from France), while compounds observed at the highest levels included coumaphos (mean concentration of 925.0 μg kg^-1^) and tau-fluvalinate (487.2 μg kg^-1^) [50]. Giroud et al. (2013) found residues of thiacloprid in 75% of their beebread samples from France; the concentrations ranged from 0.03 to 177 μg kg^-1^, in line with our findings (3.3–61.3 μg kg^-1^) [51]. Kasiotis et al. (2014) found residues of thiamethoxam, clothianidin, imidacloprid and chlorpyrifos in 29 out of 43 of their honeybee samples acquired in 2011 and 2013 [35].

Lambert et al. (2013) found residues of at least one pesticide in 72.3% (of 141 bee samples), 95.7% (of 141 honey samples), and 58.6% (of 128 bee pollen samples); these samples were acquired in 2008 and 2009 [52]. In that study 23 different pesticide active ingredients were identified in pollen. At least two residues were found in 30.5% of samples; the maximum number of different residues found in a single sample was seven. Carbendazim and amitraz were most frequently identified in pollen. The maximum absolute pesticide concentrations found in pollen were: 3.67 mg kg^-1^ (thiophanate-methyl) and 2.60 mg kg^-1^ (carbendazim). Carbendazim is a known metabolite of thiophanate-methyl and its presence in samples might be linked to the field use of the latter compound. All samples contaminated by thiophanate-methyl contained also carbendazim.

Genersch et al. (2010) found residues of 42 different pesticide active ingredients in more than 70% of their bee pollen samples acquired between 2005–2007 in Germany [53]. Coumaphos, boscalid and thiacloprid were among the most frequently identified insecticides and fungicides. Rennich et al. (2012) found coumaphos in 39.4% of their 99 samples of bee pollen acquired in the US, tau-fluvalinate in 38.4% of samples, thymol and amitraz each in 27.3% of samples, chlorpyrifos in 20.2% of samples [54]. Bernal et al. (2010) found residues of pesticides in 42% of pollen samples collected in Spain during the spring, but only in 31% of samples collected during the autumn [55]. Tau-fluvalinate and chlorfenvinphos were the most frequently detected residues.

Residues of DDE (DDT breakdown product) above LOQ values were detected in five out of 53 bee pollen samples analyzed in this study (despite the fact that DDT use in agriculture is banned in majority of developed countries, including Poland). DDE is quite commonly found in food and environmental samples; it is a POP and a legacy pesticide. Contamination with persistent legacy compounds like DDT/ DDE results mostly from long-range aerial transport of those compounds or contact with contaminated dust/solid particles.

Pesticide residues might find their way to bee pollen through drenched plants, via direct spraying of flowering crops with pesticide-containing aerosols, from pesticide pre-treated seeds, or pesticide-contaminated particles created during drilling of pesticide-treated seeds. Tau-fluvalinate is registered in some European countries as a chemical used to fight Varroa jacobsoni. Balayannis (2001) reported residues of coumaphos and tau-fluvalinate in apiculture products after the chemicals were applied in beehives to fight the latter pests [56]. However, tau-fluvalinate is also registered as a chemical used to fight various pests in crops; therefore its residues might be traced to at least two different origins.

Contamination of bee pollen might be determined by plant species foraged by bees and agricultural practices when plant protection products are applied to crops cultivated around apiaries. Some plants that require intensive chemical protection might be an important source of exposition to pesticides if foraged by bees. Bees may collect contaminated pollen [12] or nectar [57] but also may get directly exposed to spray droplets during the application of these pesticides, or dust particles during seed-dressing operations [13,17]. Direct exposition via spray might be significant if pollinator poisoning prevention rules are not observed (e.g., bloom spraying during daytime). Our pollen samples came from many species of plants foraged by bees, with a considerable share of oil seed rape pollen. According to beekeepers running apiaries from which samples with the greatest observed concentrations of tebuconazole and thiacloprid came, oil seed rape was cultivated near their apiaries. Both compounds are recommended to protect oil seed rape and are commonly applied in combined spray applications. Tebuconazole is additionally used as a seed coating, while no thiacloprid formulations are registered for this purpose in Poland. Their molecules have to show hydrolytic, oxidative, and metabolic stability to be persistent in pollen. Therefore, oil seed rape might generally be a significant source of exposure of honey bees to pesticides.

It has been well demonstrated that some ergosterol biosynthesis inhibitors (like tebuconazole fungicides) co-applied with neonicotinoids could synergistically enhance the toxicity of thiacloprid to bees because they may inhibit detoxicative cytochrome P450 monooxygenase. This phenomenon was also confirmed for several other pesticide combinations [58–60]. Synergism levels are dose and compound dependent and might be lower under realistic exposure levels and field conditions [60]. Nevertheless, EC Regulation 1107/2009 identified tebuconazole and several other substances found in bee pollen samples analyzed in this study as candidates for substitution [61].

Pohorecka et al. (2012) reported residues of neonicotinoid in bee products (including pollen) when the compounds were used for oil rape seed treatment. In addition, the resulting residues formed through this mechanism with respect to the compounds found in this study are generally in low μg kg^-1^ range [38]. However, Pohorecka et al. (2012) report that some rather high levels of neonicotinoid residues were observed when the compounds were used as a foliar spray [38]. In addition, they noted that the level of thiacloprid in bee pollen varied greatly (2.0–369.0 μg kg^-1^) despite a similar crop spraying time; in samples of pollen from orchard plants, the thiacloprid mean concentration was as high as 646 μg kg^-1^. Acetamiprid and thiacloprid were found in approx. 50 and 60% of their samples, respectively. They suggested that the disparate levels simply reflected the irregular crop coverage by the spraying solution.

Pollen is a significant component of the honeybee diet [62], it significantly determines the proper development of the brood, conditions of individual bees, and in consequence welfare of whole bee colonies. Bee castes use different food sources. Pollen is the main food of nurse honeybees, but is also used to produce royal-jelly that feeds the larvae and the queen. The foragers do not consume pollen, only nectar, and the differentiation from nurses to foragers happens after ten days in honey bees; wild bee species have different life cycles. Life expectancy of bees changes throughout the year. Worker bees live approximately 38 days in summer and up to six months in winter. According to the literature, a single colony may use for its own needs from 13.4 kg [63] up to 55 kg [64] of pollen annually. The average size of a colony may range from 20–30 to 70–100 thousand bees (along with seasonal changes in population) [65]. To evaluate the health risk to bees associated with contaminated pollen, only a conservative scenario where pesticide residues do not degrade in pollen stored in the hive and using a nurse bees as a model (showing the highest pollen consumption rates at 9.6 mg day^-1^) calculated for winter months was adopted [62,66]. At the maximum concentration of thiacloprid observed in the studied pollen samples, 136 μg kg^-1^, a bee would consume 1.3 ng of thiacloprid daily. Total lifelong intake through contaminated pollen would result in 235 ng bee^-1^. Taking into account that the thiacloprid LD50 lethal dose is approximately 17.32 μg bee^-1^ (oral) [67], it seems that thiacloprid taken (during a relatively long period) with pollen in amount of 1% of the LD50 value does not cause any acute toxicity to bees. On the other hand, the LD50 value for chlorpyrifos (bee, oral) is only approx. 0.12 μg bee^-1^ [68]. At the maximum concentration of chlorpyrifos observed in the studied pollen samples, 40 μg kg^-1^, an average nurse worker bee would consume 0.069 μg (0.384 ng a.i. daily) i.e., as much as 57% of the LD50. The worst case scenario was adopted for these calculations, taking into account the highest observed pesticide concentrations and the highest life expectancies. However, such an estimation provides a theoretical upper limit of intake values that are unlikely to be achieved under real life conditions. It must be emphasized that: (i) such estimations are fraught with considerable uncertainty due to the variable colony size/bee lifetime/pesticide profiles/pollen intake and/or bee susceptibility to various chemicals present in diet; (ii) LD50 values for the most toxic neonicotinoids (clothanidin 0.00379 μg bee^-1^, thiamethoxam 0.005 μg bee^-1^, imidacloprid 0.0037 μg bee^-1^) are more than three orders of magnitude lower than respective values for thiacloprid (17.32 μg bee^-1^) or acetamiprid (14.53 μg bee ^-1^) [55]. In addition, bees consume significantly more nectar than pollen and this might have a considerable impact on the total intake value. According to the data reported by Codling et al. (2016) nectar would contribute 20–100% of the NIs dietary intake [66]. It must also be stressed that such statements refer only to possible acute toxicity of pesticides to bees. Chronic exposure to sub-lethal doses of neonicotinoids could alter insect physiology and induce neurobehavioral changes indirectly causing their death [7,69,70]. In this respect, data on acute toxicity without information on the effects caused by chronic exposure are not sufficient to thoroughly characterize the influence of those compounds on bees. Such evaluations have been performed in other studies [70–72]. As reported by other authors, even minimal residue levels could affect pollinator health in the long term, therefore no safe exposure level can be set even at sub-lethal doses [66]. A bee queen that is expected to live 3–5 years is potentially the most significantly exposed to pesticide residues even if she feeds on royal jelly which is a “processed” product and is known to be significantly less contaminated by pesticides [62]. However, Williams et al. (2015) have recently reported that honeybee queens are severely affected by NIs at environmentally realistic concentrations and have proposed that this phenomenon might be the main driver of the honeybee colony losses [20].

Apiary products are becoming more and more popular among general populations as food products; therefore pesticide residues should be considered in respect to maximum residue levels (MRL) set according to the European Commission Regulation 396/2005 [73]. These levels were exceeded in approx. 28% of our samples. Pesticides above MRL included: prothioconazole (3 samples), tetraconazole (1), thiacloprid (6), tebuconazole (2), deltamethrin (1), bifenthrin (1), metamitron (1). According to the Sanco Pesticides Database, the MRL values for honey and other apiculture products for those compounds are set at 50, 20, 200, 50, 30, 10 and 10 μg g^-1^, respectively.

### PCBs / PBDEs

EU indicator PCBs (EC6 congeners # 28, 52, 101, 138, 153, 180) and dioxin-like PCBs (EC12 congeners # 77, 81, 105, 114, 118, 123, 126, 156, 157, 167, 169, 189) in pollen samples are summarized in Table 3. Average concentrations of EC6/EC12 PCBs (approx. 194/74 pg g^-1^, respectively) are generally in line with previously published data for various plant-origin products [74]. That might be expected taking into account that bees collect pollen just from plant flowers, and in this respect a contamination level/profile is unlikely to be significantly modified by the bees themselves.

**Table 3:** Concentrations of PCBs/PBDEs found in the analyzed samples and fractions of PCB/PBDE-positive samples.

Compound	Mean	SD	Median	MIN	MAX	Fraction		Compound	Mean	SD	Median	MIN	MAX	Fraction
	[pg g^-1^]					[%]			[pg g^-1^]					[%]
CB28	61.3	24.6	60.7	0.0	121.1	98.1		BDE 1	0.2	0.6	0.0	0.0	3.9	7.5
CB52	15.9	8.4	15.2	0.0	38.6	98.1		BDE 2	0.3	0.8	0.0	0.0	4.2	20.8
CB101	35.8	19.2	36.0	4.4	96.5	100.0		BDE 3	0.2	0.7	0.0	0.0	3.3	20.8
CB138	36.7	17.3	34.2	11.9	114.6	100.0		BDE 7	0.0	0.1	0.0	0.0	0.8	3.8
CB153	33.7	22.3	31.9	1.4	111.5	100.0		BDE 8	N/D					0
CB180	11.0	6.2	10.5	0.6	36.7	100.0		BDE 10	N/D					0
CB194	1.1	1.8	0.6	0.0	12.4	73.5		BDE 11	N/D					0
**EC6**	**194.3**	**79.1**	**187.6**	**34.3**	**413.2**	**100.0**		BDE 12	N/D					0
								BDE 13	N/D					0
CB77	7.8	3.6	7.1	3.4	23.1	100.0		BDE 15	0.0	0.2	0.0	0.0	1.2	7.5
CB81	0.8	1.1	0.6	0.2	8.1	100.0		BDE 17	0.2	0.8	0.0	0.0	3.7	15.1
CB105	1.6	1.8	1.2	0.0	10.5	83.0		BDE 25	N/D					0
CB114	17.6	14.7	13.2	1.2	83.3	100.0		BDE 28+33	0.9	2.3	0.0	0.0	13.0	45.3
CB118	37.7	26.3	32.0	11.1	178.1	100.0		BDE 30	N/D					0
CB123	1.8	1.5	1.7	0.0	10.9	98.1		BDE 32	0.1	0.3	0.0	0.0	2.1	5.7
CB126	0.0	0.0	0.0	0.0	0.2	20.8		BDE 35	0.04	0.3	0.0	0.0	1.6	1.9
CB156	2.9	1.9	2.5	0.5	11.2	100.0		BDE 37	0.04	0.3	0.0	0.0	1.6	1.9
CB157	1.2	2.0	0.6	0.0	10.8	98.1		BDE 47	3.0	3.1	1.9	0.0	12.5	94.3
CB167	1.9	2.2	1.3	0.3	13.3	100.0		BDE 49	0.5	1.6	0.0	0.0	9.5	43.4
CB169	0.4	1.3	0.2	0.0	9.1	0.8		BDE 66	0.4	0.9	0.0	0.0	4.7	45.3
CB189	0.6	1.3	0.4	0.0	9.7	96.2		BDE 71	0.2	0.4	0.0	0.0	1.7	28.3
**EC12**	**74.4**	**49.8**	**59.8**	**20.2**	**312.7**	**100.0**		BDE 75	3.1	5.3	0.0	0.0	17.0	52.8
								BDE 77	N/D					0
								BDE 85	0.7	3.9	0.0	0.0	24.0	17.0
								BDE 99	2.7	6.2	0.7	0.0	34.6	86.8
								BDE 100	1.4	4.8	0.4	0.0	29.4	71.7
								BDE 116	N/D					0
								BDE 118	2.1	7.2	0.0	0.0	32.1	17.0
								BDE 119	0.5	2.4	0.0	0.0	14.6	22.6
								BDE 126	0.04	0.2	0.0	0.0	1.5	9.4
								BDE 138	0.4	1.2	0.0	0.0	5.2	47.2
								BDE 153	0.6	1.8	0.0	0.0	10.5	41.5
								BDE 154	0.9	1.7	0.0	0.0	7.5	60.4
								BDE 155	0.02	0.1	0.0	0.0	0.5	9.4
								BDE 166	N/D					0
								BDE 181	0.1	0.3	0.0	0.0	1.2	32.1
								BDE 183	1.4	1.8	0.8	0.0	6.2	73.6
								BDE 190	0.1	0.3	0.0	0.0	1.6	26.4
								**∑ BDE 39**	**20.3**	**27.7**	**12.7**	**0.0**	**156.0**	**98.1**

Literature data on PCBs levels in apiary products are limited. Erdogrul et al. (2007) reported the mean concentration of EC6 + CB 118 in Turkish honey samples at 1.48 ng g^-1^ [75]. Of the 111 honey samples analyzed by Herrera et al. (2005), 83% were PCB-positive, and concentrations of individual EC6 PCBs were within the 4–593 ng g^-1^ range.

**Fig 3:**
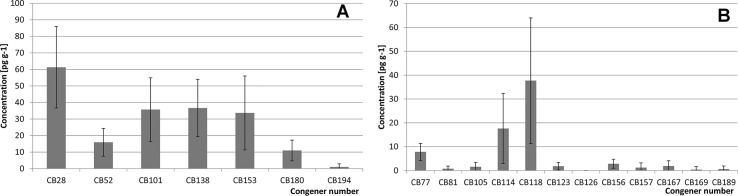
Indicator (A) and dioxin-like (B) chlorinated biphenyls profiles observed in the analyzed pollen samples.

CBs profiles observed in our bee pollen samples were typical for plant origin products: lower chlorinated congeners dominated. Profile of EC6 congeners is shown in Fig 3.

Congener #28 was the most common member of the profile (61 pg g^−1^ average concentration, 31% average relative share in the profile). Other relatively abundant congeners included: #101 (35 pg g^−1^, 18%), #138 (36 pg g^−1^, 19%), and #153 (33 pg g^−1^, 17%). The profile most likely reflects the distribution/transportation of individual substances within the environment, including long-range aerial transport, accumulation on particulate matter, and/or accumulation on plant surfaces. Processes of degradation of some congeners via de-chlorination could modify the profile with respect to the profiles observed in animal-origin products. The share of heavier congeners in animal tissues might be increased with respect to plant-origin feeds due to bio-accumulation processes (see discussion in [47,74].

Erdogrul et al. (2007) reported CB congeners # 28 and 52 while Herrera et al. (2005) reported CB congeners # 28 and 138 as the major PCBs in their honey samples; other EC6 congeners were identified at lower frequencies and concentrations [28,75]

The profile of 12 dioxin-like PCBs found in our pollen samples is shown in Fig 3. The three congeners found at the highest concentrations included #114, 118, and 77 (average concentrations were 17.6, 37.6, and 7.8 pg g^−1^, relative shares in the profile were 23%, 50%, and 11%, respectively).

**Fig 4:**
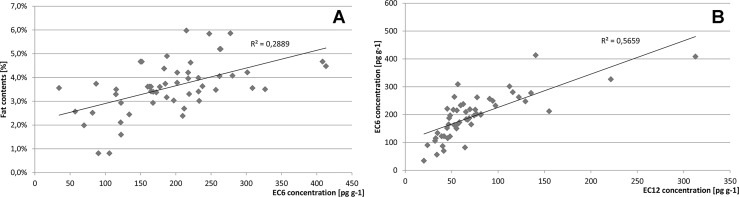
Relationship between lipid contents and EC6 concentrations (A) and between EC6 and EC12 concentrations (B) in the analyzed samples of bee pollen.

Most of POPs tend to accumulate in fat due to their lipophilic characteristics and partition coefficients. In most cases, such accumulation would result in a high correlation between POP concentrations and lipid contents in the matrix (in our pollen samples lipid contents varied from 0.81% to 5.98% with a mean concentration of 3.61%). However, no significant correlation was observed in this case; the coefficient of determination R^2^ was only 0.29, see Fig 4A.

The major path of contamination i.e., precipitation from air might explain the lack of any stronger correlation: PCBs most likely do not penetrate pollen particles and are located on their surface only. In addition, our pollen samples represented plants of different susceptibility to contamination resulting from differences in plant architecture.

A significantly higher correlation was observed between indicator (EC6) and dioxin-like (E12) PCB congeners, see Fig 4B. This reflects similar sources of contamination, only potentially modified by some degradation processes running in the environment.

Generally, levels of BDE congeners measured in our samples were in the low pg g^-1^ range, see Table 3. Average concentrations of all 39 BDEs (Σ39 BDE) were 20.3±27.7 pg g^−1^. The concentrations were significantly less than levels reported in the literature, in which neutral and lipophilic contaminants (such as BFRs) are (bio-) accumulated. The concentrations are generally in line with those observed previously in plant-origin products.

Literature data on BDEs in apiary and plant-origin products are sparse. Erdogrul et al. (2007) did not detect any BDEs in honey samples above LOD (LOD was 20 pg g^-1^) [75]. Of the 35 honey samples analyzed by Mohr et al. (2014), 91% were BDE-positive, average concentration of sum of 15 evaluated congeners was 1.2 pg g^-1^ [29]. A rather high variability of concentrations (0.18–1.39 pg g^-1^) was attributed to a multitude of sample origins. Contrary to that, Wang et al. (2010b) reported high PBDE levels in honey sampled in various geographical regions of the world, from 300 to 10,550 pg g^-1^ depending on sample origin [76]. Such levels are typical for high-lipid products like fish generally known as a relatively highly contaminated food. Higher contamination levels in honey sampled were noted in developed countries than those in samples from developing countries or regions (indicated by the authors as “background”), but the differences were not statistically evident. No explanation for the high PBDE levels in honey was proposed.

**Fig 5:**
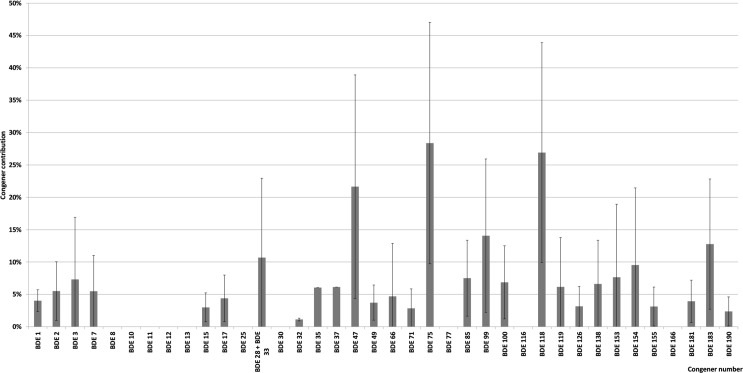
Profile of congeners found in BDE-positive bee pollen samples.

The average profile of congeners found in our BDE-positive pollen samples is shown in Fig 5. The profile was dominated by congeners #75, 47, and 99 (3.1, 3.0, and 2.7 pg g^−1^, respectively). Congeners #118, 100, and 183 were somewhat less common (2.1, 1.4, 1.4 pg g^-1^, respectively). Of the other BDEs, none exceeded 1 pg g^-1^. Quite different profiles have been reported for animal- or plant-origin products, where the range of dominating congeners is usually much narrower (reflecting composition of BDEs technical mixtures). Our results are generally in line with those reported by Mohr et al. (2014) and by Wang et al. (2010b), who found congeners #47/99 and #47/138/153 (respectively) as the most common ones [29,76]. Some geographical variability of the observed BDEs profiles has also been noted, although this was not observed for PCBs.

Levels and profiles of PBDEs depend on paths along which they are transformed within the environment. BDEs might degrade relatively easily, forming a wide spectrum of lower-brominated analogs (tri- to hepta-substituted). That might (at least partially) explain the observed variability/complexity of PBDEs profiles and generally low levels of these compounds in apiary matrices. The influence of surface nature of contamination and relatively low lipid contents might in this case be of particular importance for plant surface contamination/decontamination (decontamination via UV radiation-catalyzed degradation). Influence of such factors on stability/profiles of PBDEs was addressed in detail in our previous report [77,78].

**Fig 6:**
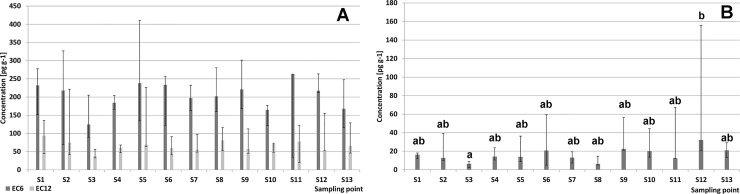
Average concentrations (medians, 25/75 percentiles) for 13 sampling locations. A (left): six indicator CBs (EC6, dark bars) and twelve dioxin-like CBs (EC12, light bars). B (right): PBDEs. Significantly different groups are marked with letters a, b, or ab (α = 0.05).

**Fig 7:**
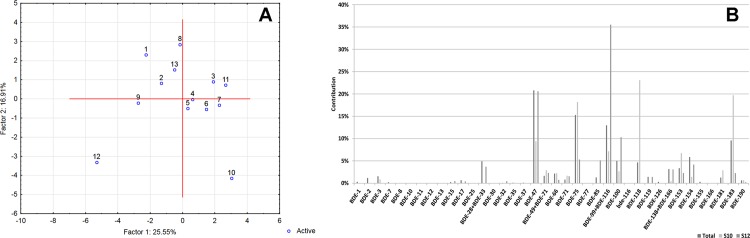
A- PCA scatter plot of PBDEs from 13 different sampling regions B–Average congener profile and the profiles observed for the most diverse sampling points identified by PCA.

Statistical assessment of data has not revealed any meaningful differences between the average concentrations of PCBs in samples acquired in the 13 different locations in Poland, but such differences have been demonstrated with respect to PBDEs. Wang et al. (2010) noted that the possibility of emission of PBDEs from various materials is still high since PBDE-containing products are still in use [76]. Jonez-Otazo et al. (2005) suggested that that dust/particulate matter could be a significant source of exposure to such compounds [79]. Significant differences marked in Fig 6B may reflect the fact that some active emission sources (municipal waste dump sites, electronic processing plants, highly urbanized areas etc.) are located near some of the involved apiaries, while no such sources are located near the others. Similar variability has already been demonstrated for other matrices such as birds [80]. As revealed by the Principal Component Analysis (PCA) scatter plots, BDE profiles were quite similar for samples acquired at 11 out of 13 sampling points (see Fig 7). A somewhat higher share of BDE 99 was observed in samples from region 12, which was contaminated to the highest degree. This might indicate that something other than an environmental background source(s) of contamination is (are) at work in that region. No significant clustering was observed for PCBs.

## Conclusions

1. Maximum residue levels set by EU regulations for pesticides in food were exceeded in 28% of the samples. Pesticides above MRL included prothioconazole (3 samples), tetraconazole (1), thiacloprid (6), tebuconazole (2), deltamethrin (1), bifenthrin (1), metamitron (1).
2. Neonicotinoide (thiacloprid) insecticides were identified in a substantial fraction of all of the positive samples.
3. Generally low PCB levels and very low PBDE levels were found in 53 analyzed bee pollen samples. Observed congener profiles suggest degradation of PBDEs, most likely related to surface-only contamination of plants through air precipitation (the most probable route of exposure).
4. The fact that there were no significant differences in congener profiles observed in samples acquired in a majority of the sampling locations suggests that there were similar contamination routes prevailing in all those locations.
